# Adjunctive berberine improves hormonal, metabolic, and inflammatory profiles in women with polycystic ovary syndrome: a retrospective case–control study

**DOI:** 10.3389/fendo.2026.1700331

**Published:** 2026-05-22

**Authors:** Lingling Gao, Yuxin Ju, Yuanyuan Zhu, Jianbo Xu, Dan Lu

**Affiliations:** 1 Department of Obstetrics and Gynecology, Northern Jiangsu People’s Hospital Affiliated to Yangzhou University, Yangzhou, Jiangsu, China; 2 Department of Obstetrics and Gynecology, Northern Jiangsu People’s Hospital, Yangzhou, Jiangsu, China

**Keywords:** berberine, hormones, inflammation, insulin resistance, polycystic ovary syndrome

## Abstract

**Background:**

Polycystic ovary syndrome (PCOS) is often associated with androgen excess, insulin resistance, and low-grade inflammation. While progesterone is standard for regulating menstrual cycles, additional therapies are often needed to address metabolic and inflammatory dysfunctions. Berberine has shown insulin-sensitizing and anti-inflammatory actions that may complement standard hormonal therapy.

**Objectives:**

This study was designed to evaluate whether adding oral berberine to luteal-phase progesterone improves anthropometric, hormonal, metabolic, and inflammatory indicators in women with PCOS compared with progesterone alone.

**Methods:**

Women with PCOS who received either berberine plus luteal-phase progesterone or progesterone alone for 3 months were retrospectively analyzed. Clinical records were reviewed for anthropometric, hormonal, metabolic, and inflammatory outcomes, assessed at baseline and after treatment.

**Results:**

The berberine group showed significant within-group reductions in weight, BMI, and WHR; no significant changes occurred in the controls. Crude post-treatment between-group differences in weight, BMI, and WHR were not significant, but became significant for weight and BMI after adjustment. Although the LH and LH/FSH ratio declined in both groups, the adjusted post-treatment values were significantly lower in the berberine group compared to the control group, indicating a superior effect. Total testosterone fell only in the berberine group, yielding significant crude and adjusted between-group differences. Metabolically, fasting insulin, FBS, and HOMA-IR decreased significantly in the berberine group but not in the control group, with crude and adjusted between-group differences remaining significant. Inflammatory markers (CRP, TNF-α, IL-6) declined significantly only in the berberine group. Between-group comparisons were significant in CRP, TNF-α, and IL-6 at 3 months after adjustment.

**Conclusions:**

These preliminary findings suggest that adjunctive berberine therapy may improve anthropometric, hormonal, metabolic, and inflammatory profiles in women with PCOS. However, well-designed randomized controlled trials are required to confirm these effects and establish definitive clinical recommendations.

## Introduction

Polycystic ovary syndrome (PCOS) is a common, heterogeneous endocrine disorder, affecting approximately 5%–10% of women in their reproductive years (1). It is characterized by oligo-ovulation or anovulation, biochemical or clinical hyperandrogenism, and polycystic ovarian morphology, which is associated with adverse reproductive outcomes across the lifespan. Beyond reproductive dysfunction, PCOS is tightly linked to metabolic derangements. A large proportion of PCOS patients exhibit insulin resistance, compensatory hyperinsulinemia, and adverse lipid profiles, which collectively increase long-term risks of type 2 diabetes and cardiovascular disease (2, 3). In addition, chronic low-grade systemic inflammation and oxidative stress have been increasingly recognized as central components of PCOS pathophysiology that interact with insulin resistance and hyperandrogenism to impair folliculogenesis and worsen cardiometabolic risk (4). Conventional symptom-oriented treatments, such as cyclic progesterone to induce withdrawal bleeding, primarily provide endometrial protection and regulate menstruation, but exert minimal effects on the underlying metabolic disturbances and chronic inflammation that characterize PCOS (5). These metabolic–inflammatory features make insulin-sensitizing and anti-inflammatory treatments attractive targets for improving both reproductive and metabolic outcomes in PCOS.

Berberine (BBR) is a natural isoquinoline alkaloid isolated from plants such as *Coptis* and *Berberis* species and has a long history of medicinal use. In patients with type 2 diabetes and metabolic syndrome, randomized and comparative clinical studies have demonstrated that berberine significantly reduced fasting glucose and insulin levels and improved lipid metabolism (6, 7), with efficacy partly comparable to metformin in some cohorts (8). The underlying mechanisms are thought to involve activation of AMP-activated protein kinase (AMPK), improvement of insulin signaling and cellular glucose uptake, and suppression of inflammatory pathways (9, 10), all of which are closely linked to the metabolic and endocrine disturbances observed in PCOS.

Preclinical and clinical studies exploring berberine in PCOS are encouraging but heterogeneous. Several randomized controlled trials and small pilot studies compared berberine with metformin or studied berberine as an adjunct to cyproterone acetate, reporting improvements in insulin sensitivity, lipid parameters, and some reproductive indices (ovulation rate, endometrial receptivity) in subsets of PCOS patients (11). One explanatory study demonstrated significant reductions in insulin resistance, inflammatory markers, triglycerides, total testosterone, body mass index (BMI), visceral adipose tissue, fat mass, and dermatologic scores related to hyperandrogenism (12). However, these findings were derived from a one-group pretest–posttest design without a control group. In another controlled study, berberine phytosome supplementation improved the resumption of regular menstruation and normalization of ovarian morphology, but did not affect the metabolic or hormonal profiles at study completion (13). While the efficacy of berberine as a monotherapy or as an alternative to metformin has been extensively investigated, its role as an adjunctive treatment in routine clinical settings remains underexplored. In clinical practice, women with PCOS frequently receive cyclic progesterone therapy to induce withdrawal bleeding, regulate menstrual cycles, and protect the endometrium. However, progesterone therapy alone does not address the underlying pathophysiological drivers of PCOS, such as insulin resistance, hyperandrogenism, and chronic low-grade inflammation. Consequently, there is a critical evidence gap regarding the potential benefits of combining berberine with standard progesterone therapy.

Given these considerations, we conducted a retrospective case–control study to evaluate whether adding oral berberine to luteal-phase progesterone therapy over 3 months improves anthropometric indices, reproductive hormones, metabolic parameters, and inflammatory biomarkers in women with PCOS, compared with progesterone alone.

## Methods

### Study design

This study was designed as a retrospective case–control study conducted in accordance with the principles of the Helsinki Declaration. The study was approved by the Ethics Committee of Northern Jiangsu People’s Hospital Affiliated to Yangzhou University, and informed consent was collected from all participants.

### Participants

Clinical records of women diagnosed with PCOS according to the 2003 Rotterdam criteria were retrospectively reviewed from the outpatient department of Northern Jiangsu People’s Hospital Affiliated to Yangzhou University between August 2024 and August 2025. Sample size calculation was performed using G*Power. Based on previous literature and our preliminary clinical observation, a large effect size was assumed (Cohen’s *d* = 0.92). The calculation was based on a two-tailed independent samples *t*-test with a significance level (*α*) of 0.05 and a desired statistical power (1 − *β*) of 0.80. The required sample size was calculated to be 24 patients per group to detect a statistically significant difference between the two groups. To account for potential data exclusion or missing records during the retrospective data collection process, we expanded the target sample size and ultimately included a total of 60 patients (30 per group), which is fully sufficient to detect a true clinical effect and minimize the risk of false-negative results. A total of 60 eligible patients were included. All women were between 18 and 40 years of age with a body mass index (BMI) of 18–35 kg/m^2^. The exclusion criteria were concomitant endocrine or metabolic disorders such as thyroid dysfunction, androgen-secreting tumors, diabetes mellitus, adrenal hyperplasia, and Cushing’s syndrome. To minimize the confounding effects, rigorous exclusion criteria were applied. Specifically, patients were excluded if they had any clinical signs or symptoms of hepatic or renal dysfunction, severe dyslipidemia requiring statin therapy, acute infections (e.g., respiratory or urinary tract infections), chronic inflammatory or autoimmune diseases, or if they had used any other hormone therapy, insulin sensitizers, or anti-inflammatory medications (such as non-steroidal anti-inflammatory drugs or corticosteroids) within the 3 months prior to enrollment or during the 3-month treatment period. Based on treatment records, patients were divided into two groups: The control group received dydrogesterone 10 mg or micronized progesterone 100 mg twice daily for 10 or 12 days during the luteal phase of each cycle. The berberine group received oral berberine hydrochloride tablets at a dosage of 500 mg three times a day for 3 months combined with oral progesterone during the luteal phase of each menstrual cycle. Patients were specifically instructed to take berberine immediately before or during meals to optimize metabolic efficacy and minimize potential gastrointestinal adverse effects. The total duration of the prescribed treatment for both groups was 3 months. Regarding treatment compliance, as this was a retrospective real-world observational study, adherence could not be evaluated through strict prospective methods such as pill counting or patient diaries. Instead, compliance was primarily assessed based on physician documentation in the electronic medical records during the regular 1- and 3-month outpatient follow-up visits, relying on patient self-reporting. Patients who explicitly reported poor adherence, discontinued the medication early, or failed to complete the 3-month follow-up were excluded from the final analysis. Due to the retrospective observational nature of this study, treatment allocation was not randomized. The rationale for choosing berberine as an adjunct to progesterone, rather than progesterone alone, was based on shared decision-making in routine clinical practice. Specifically, berberine supplementation was proposed to patients who exhibited insulin resistance, obesity, or hyperandrogenic symptoms, and who either preferred phytotherapeutic alternatives or were reluctant to initiate standard insulin-sensitizing agents (such as metformin). Patients who declined the adjunct therapy or preferred standard monotherapy received progesterone alone. Clinical and laboratory data were extracted at two time points: baseline (before treatment initiation) and after 3 months of therapy.

### Outcomes

Anthropometric outcomes included body weight (kg), BMI (kg/m²), and waist–hip ratio (WHR), which were recorded in the medical charts using standardized procedures. The formulation (weight in kg/height in m^2^) was used to calculate BMI. Systolic blood pressure (SBP) and diastolic blood pressure (DBP) were also collected. Hormonal parameters included serum luteinizing hormone (LH), follicle-stimulating hormone (FSH), LH/FSH ratio, and total testosterone. Metabolic outcomes comprised fasting insulin, fasting blood sugar (FBS), and homeostatic model assessment of insulin resistance (HOMA-IR), while inflammatory markers included C-reactive protein (CRP), tumor necrosis factor-alpha (TNF-α), and interleukin-6 (IL-6). All variables were measured at baseline and again after 3 months of treatment. Safety data and adverse events, including gastrointestinal reactions, dizziness, and any incidence of abnormal liver or kidney function, were retrospectively extracted from the electronic medical records and outpatient follow-up notes.

### Statistical analysis

The normality of the data distribution was evaluated using the Shapiro–Wilk test. Continuous variables were presented as mean ± standard deviation (SD) for normally distributed variables. An independent-samples *t*-test was used to compare the continuous variables between the berberine and control groups. If the data were not normally distributed, the Mann–Whitney *U* test was used. Within-group changes (pre–post changes) over 3 months were assessed using paired *t*-tests or the Wilcoxon signed-rank tests for each group. Analysis of covariance (ANCOVA) was used for between-group comparison at 3 months by adjustment on pre-treatment results. Before conducting the ANCOVA, the underlying statistical assumptions were rigorously evaluated. The linearity between the covariate (baseline values) and the dependent variable (post-treatment outcomes) was confirmed visually using scatterplots. Additionally, the assumption of homogeneity of regression slopes was tested and met, as no significant interactions between the treatment group and the baseline covariates were observed (all *p* > 0.05). Homogeneity of variances was verified using Levene’s test. All statistical analyses were performed using Stata version 15.0. A *p*-value (two-tailed) less than 0.05 was considered statistically significant.

## Results

### Baseline characteristics

At baseline, there were no significant differences between the berberine plus progesterone group and the progesterone-only control group in age, height, weight, BMI, WHR, SBP, or DBP (*p* > 0.05 for all) (**Table 1**). This indicates that the two groups were comparable before the treatment.

**Table 1:** Baseline characteristics of the study participants.

Baseline characteristics	Berberine (*n* = 30)	Control (*n* = 30)	*P*-value
Age (years)	27.30 ± 4.35	28.07 ± 4.59	0.5094
Height (cm)	163.47 ± 4.26	162.63 ± 4.16	0.4465
Weight (kg)	66.07 ± 8.88	64.43 ± 10.13	0.5092
BMI (kg/m^2^)	24.76 ± 3.56	24.37 ± 3.79	0.6814
WHR	0.82 ± 0.04	0.82 ± 0.05	0.7630
SBP	124.50 ± 5.62	124.57 ± 6.71	0.9669
DBP	79.5 ± 6.88	80.83 ± 6.44	0.4414

All values are mean ± SD. Statistical significance is based on an independent *t*-test.

BMI, body mass index; WHR, waist-to-hip ratio; SBP, systolic blood pressure; DBP, diastolic blood pressure.

### Anthropometric outcomes

After 3 months of treatment, the berberine group showed significant within-group reductions in weight, BMI, and WHR. In contrast, the control group showed no significant changes in these parameters. Crude post-treatment between-group differences in weight, BMI, and WHR were not statistically significant. However, after adjustment for age, height, SBP, DBP, and baseline values using ANCOVA, between-group differences at 3 months were statistically significant for weight and BMI (*p* < 0.001), but not for WHR (**Table 2**).

**Table 2:** Post-treatment anthropometric characteristics of women with PCOS.

Anthropometric characteristics	Berberine (*n* = 30)	Control (*n* = 30)	*p*-value^a^	Adjusted *p*-value^b^
Weight (kg)				
Baseline	66.07 ± 8.88	64.43 ± 10.13	0.5092	–
Endpoint	61.95 ± 7.41	64.40 ± 9.82	0.2799	<0.001*
*p*-value^c^	<0.001*	0.7122	–	–
BMI (kg/m^2^)				
Baseline	24.76 ± 3.56	24.37 ± 3.79	0.6814	–
Endpoint	23.22 ± 2.98	24.36 ± 3.68	0.1912	<0.001*
*p*-value^c^	<0.001*	0.7235		–
WHR				
Baseline	0.8223 ± 0.0421	0.8143 ± 0.0466	0.7630	–
Endpoint	0.8196 ± 0.0387	0.8187 ± 0.0511	0.6315	0.570
*p*-value^c^	0.0182*	0.2054		–

All values are mean ± SD.

^a^ Between-group *p*-values.

^b^ Between-group *p*-values based on ANCOVA adjusted for age, height, SBP, DBP, and baseline values of each variable.

^c^ Within-group *p*-values.

*Statistically significant (*p* < 0.05).

### Hormonal profile

In the berberine group, serum LH decreased markedly (*p* < 0.001), whereas the control group also showed a decrease (*p* < 0.001). The crude and adjusted post-treatment between-group values remained significantly different between the two groups (*p* = 0.0045; *p* < 0.001). FSH levels remained unchanged in both groups. The LH/FSH ratio significantly decreased both in the berberine group and the control group. After controlling for important factors including age, BMI, SBP, DBP, and the baseline values of each variable as covariates, the LH/FSH ratio showed a significant post-treatment difference between the two groups with an adjusted *p* = 0.012. Testosterone levels significantly decreased in the berberine group, while the control group showed no significant change. The crude and adjusted between-group differences were both significant (*p* < 0.001) (**Table 3**).

**Table 3:** Post-treatment hormonal profile of women with PCOS.

Hormonal profile	Berberine (*n* = 30)	Control (*n* = 30)	*p*-value^a^	Adjusted *p*-value^b^
LH (mIU/mL)				
Baseline	14.05 ± 6.21	13.30 ± 5.30	0.6158	–
Endpoint	7.87 ± 0.77	8.59 ± 1.08	0.0045*	<0.001*
*p*-value^c^	<0.001*	<0.001*	–	–
FSH (mIU/mL)				
Baseline	6.87 ± 1.47	6.90 ± 1.72	0.9281	–
Endpoint	6.67 ± 0.97	6.82 ± 1.48	0.6380	0.307
*p*-value^c^	0.1652	0.3256		–
LH/FSH				
Baseline	2.09 ± 0.90	2.01 ± 0.85	0.7210	–
Endpoint	1.19 ± 0.16	1.30 ± 0.26	0.0652	0.012*
*p*-value^c^	<0.001*	<0.001*		–
T (mIU/mL)				
Baseline	1.96 ± 0.40	1.89 ± 0.43	0.5513	
Endpoint	1.44 ± 0.26	1.86 ± 0.41	<0.0001*	<0.001*
*p*-value^c^	<0.001*	0.1185		

All values are mean ± SD.

^a^ Between-group *p*-values.

^b^ Between-group *p*-values based on ANCOVA adjusted for age, BMI, SBP, DBP, and baseline values of each variable.

^c^ Within-group *p*-values.

*Statistically significant (*p* < 0.05).

### Metabolic profile

The berberine group exhibited significant reductions in fasting insulin, FBS, and HOMA-IR. The control group did not show significant changes in these metabolic indices. The crude and adjusted between-group differences at 3 months remained significant for fasting insulin, FBS, and HOMA-IR after ANCOVA adjustment for age, BMI, SBP, DBP, and baseline values (**Table 4**).

**Table 4:** Post-treatment metabolic profile of women with PCOS.

Metabolic profile	Berberine (*n* = 30)	Control (*n* = 30)	*p*-value^a^	Adjusted *p*-value^b^
Fasting insulin (μU/mL)				
Baseline	11.47 ± 3.26	10.90 ± 4.24	0.5610	–
Endpoint	8.96 ± 2.24	10.82 ± 3.67	0.0209*	<0.001*
*p*-value^c^	<0.001*	0.6261	–	–
FBS (mmol/L)				
Baseline	5.32 ± 0.52	5.35 ± 0.51	0.8338	
Endpoint	5.08 ± 0.34	5.38 ± 0.43	0.0035*	<0.001*
*p*-value^c^	0.0023*	0.2995		
HOMA-IR				
Baseline	2.72 ± 0.86	2.64 ± 1.18	0.7609	
Endpoint	2.15 ± 0.60	2.63 ± 1.03	0.0322*	<0.001*
*p*-value^c^	<0.001*	0.7299		

All values are mean ± SD.

HOMA-IR, homeostatic model assessment of insulin resistance; FBS, fasting blood sugar.

^a^ Between-group *p*-values.

^b^ Between-group *p*-values based on ANCOVA adjusted for age, BMI, SBP, DBP, and baseline values of each variable.

^c^ Within-group *p*-values.

*Statistically significant (*p* < 0.05).

### Inflammatory markers

Following the 3-month intervention, the berberine group demonstrated significant reductions in all measured systemic inflammatory markers, including CRP, TNF-α, and IL-6. The control group did not show significant changes in CRP, TNF-α, or IL-6. Crude between-group differences in CRP, TNF-α, and IL-6 were not statistically significant. However, after adjustment for age, height, SBP, DBP, and baseline values, the adjusted between-group comparisons revealed significant differences in CRP, TNF-α, and IL-6 after 3 months of treatment (*p* < 0.001) (**Table 5**).

**Table 5:** Post-treatment inflammatory cytokines of women with PCOS.

Inflammatory cytokines	Berberine (*n* = 30)	Control (*n* = 30)	*p*-value^a^	Adjusted *p*-value^b^
CRP				
Baseline	3.15 ± 0.62	2.96 ± 0.49	0.1933	
Endpoint	2.71 ± 0.41	2.93 ± 0.48	0.0593	<0.001*
*p*-value^c^	<0.001*	0.2478		–
TNF-α				
Baseline	7.41 ± 2.76	7.46 ± 2.90	0.9487	
Endpoint	6.21 ± 2.06	7.31 ± 2.28	0.0549	<0.001*
*p*-value^c^	<0.001*	0.3315		
IL-6				
Baseline	3.58 ± 0.43	3.53 ± 0.46	0.6215	
Endpoint	3.38 ± 0.31	3.55 ± 0.37	0.0550	<0.001*
*p*-value^c^	<0.001*	0.6131		

All values are mean ± SD.

^a^ Between-group *p*-values.

^b^ Between-group *p*-values based on ANCOVA adjusted for age, BMI, SBP, DBP, and baseline values of each variable.

^c^ Within-group *p*-values.

*Statistically significant (*p* < 0.05).

### Safety and adverse events

Overall, the adjunctive berberine therapy was well tolerated. During the 3-month treatment period, no severe adverse events were recorded in either group, such as significant liver or kidney dysfunction, severe allergic reactions, or events requiring hospitalization. In the berberine group, three patients (10%) reported mild gastrointestinal symptoms, including transient nausea, mild constipation, or abdominal discomfort. These gastrointestinal reactions were generally self-limiting, mostly occurred during the first 2 weeks of treatment, and were effectively managed by ensuring the medication was taken immediately after meals. No patients discontinued the berberine treatment due to these mild adverse effects. In both groups, four patients reported mild breast tenderness or mild dizziness, which are known, predictable effects of cyclic progesterone.

## Discussion

This 3-month, controlled study showed that adding berberine to progesterone produced consistent improvements across anthropometric, endocrine, metabolic, and inflammatory domains in women with PCOS. Within-group reductions in weight and BMI were accompanied by significant between-group advantages after covariate adjustment. While both groups exhibited reductions in LH and LH/FSH, only the berberine group showed greater decreases in total testosterone levels. Furthermore, insulin resistance (fasting insulin, FBS, HOMA-IR) and inflammatory markers (CRP, TNF-α, IL-6) improved robustly versus progesterone alone. It is noteworthy that for certain outcomes (e.g., weight, BMI, and inflammatory markers), crude between-group differences were not statistically significant but reached significance after ANCOVA adjustment. This shift occurs because crude analyses only compare post-treatment values, ignoring baseline variations. By incorporating baseline values as covariates, ANCOVA effectively reduces the error variance associated with initial individual disparities, thereby increasing the statistical power to detect the true isolated effect of the berberine intervention. All of these indicated that berberine is very useful as an adjunctive treatment for PCOS.

Anthropometric measures (weight, BMI, WHR) improved significantly within the berberine group. The between-group differences of weight and BMI were also statistically significant before and after ANCOVA adjustment. However, the WHR was reduced only after ANCOVA adjustment. Berberine’s primary pharmacological action is centered on insulin sensitization and metabolic regulation, and its direct efficacy in altering body fat distribution (central adiposity) may be inherently weak without concurrent intensive lifestyle modifications. Moreover, as a real-world outpatient study, patient lifestyle factors, such as uncontrolled dietary caloric intake and varying physical activity levels, were not strictly standardized. These unmeasured confounding variables likely played a dominant role and may have masked any subtle anthropometric benefits of berberine.

The hormonal changes observed in our study, with a significant decline in total testosterone, reduction in LH and the LH/FSH ratio, and no meaningful change in FSH, are consistent with prior clinical evidence linking berberine’s insulin-sensitizing actions to improvements in ovarian androgen excess. Interestingly, our results demonstrated a significant reduction in serum LH levels in both the control and berberine groups after treatment, but the adjunctive berberine therapy achieved a markedly superior suppression of LH hypersecretion compared to progesterone monotherapy. While exogenous progesterone provides the fundamental endocrine correction by restoring negative feedback on the hypothalamic–pituitary–ovarian (HPO) axis and suppressing the rapid pulsatile GnRH release and subsequent LH hypersecretion, berberine confers a crucial synergistic effect. This additional effect is intrinsically linked to berberine’s metabolic regulation. In the pathophysiology of PCOS, hyperinsulinemia and systemic inflammation directly stimulate pituitary LH synthesis and amplify ovarian androgen production. By effectively reversing insulin resistance and reducing systemic inflammation, berberine eliminates these metabolic drivers, which amplify LH pulse amplitude. Therefore, acting in tandem with progesterone, berberine actively enhances endocrine regulation, providing a dual-target approach that more comprehensively normalizes LH levels than progesterone alone. Several clinical studies have reported that oral berberine reduced serum total testosterone and free androgen index and raised sex hormone-binding globulin (SHBG) in women with PCOS, often in parallel with improvements in insulin resistance and HOMA-IR (14). The mechanism is thought to involve improvements in insulin sensitivity, leading to reduced insulin-stimulated ovarian androgen production (15).

In terms of metabolic effects, berberine significantly lowered fasting insulin, FBS, and HOMA-IR in our study, both before and after adjusting for baseline values and covariates. Previous studies suggested that berberine activated AMPK by increasing the cellular AMP/ATP ratio, an effect attributed to partial inhibition of mitochondrial respiratory complex I, thereby promoting glucose uptake and improving insulin sensitivity *in vitro* and *in vivo* (16, 17). In parallel, berberine is thought to upregulate insulin receptor (INSR) expression via protein kinase C-dependent pathways, enhancing insulin signaling at the receptor level (18, 19). Together, it is speculated that AMPK activation and INSR upregulation provide convergent routes to the observed reductions in fasting insulin and HOMA-IR, and secondarily may attenuate ovarian androgen production driven by hyperinsulinemia. Preclinical PCOS models further support our endocrine and metabolic results. In letrozole-induced PCOS rats, berberine restored ovarian morphology and improved insulin resistance by upregulating GLUT4 through PI3K/AKT activation while dampening MAPK signaling (20). These pathway effects align with our clinical observations of improved metabolic profiles.

While our clinical findings robustly align with the established metabolic and anti-inflammatory properties of berberine, it is important to note that we did not directly evaluate intracellular molecular markers in this cohort. Therefore, the mechanistic explanations discussed herein, specifically regarding the modulation of the AMPK, PI3K/AKT, and NF-κB signaling pathways, should be strictly interpreted as hypothesis-generating. These proposed molecular mechanisms provide a plausible biological rationale for the clinical improvements observed in our study, but they warrant further validation in targeted experimental models or prospective translational trials.

Notably, our study also found marked reductions in CRP, TNF-α, and IL-6 levels after berberine supplementation, suggesting potent anti-inflammatory effects. While previous protocols have established the basic metabolic benefits of berberine, a primary novelty of our study lies in its targeted evaluation of systemic inflammatory markers (e.g., CRP, TNF-α, IL-6). Chronic low-grade inflammation is increasingly recognized as a key feature of PCOS, contributing to insulin resistance and cardiovascular risk (21). By demonstrating that adjunctive berberine significantly ameliorates this inflammatory status alongside standard progesterone therapy, our findings break beyond the traditional framework of purely metabolic or endocrine regulation. Across cellular and animal systems, berberine’s anti-inflammatory actions may involve NF-κB pathway inhibition and modulation of pro-inflammatory cytokine expression (22, 23). The significant declines in CRP, TNF-α, and IL-6 in our berberine group may in turn relieve cytokine-mediated insulin resistance and steroidogenic dysregulation in PCOS. This provides novel, multidimensional clinical evidence supporting berberine’s pleiotropic role in breaking the pathological vicious cycle of PCOS.

Clinically, the combined improvement in insulin resistance, inflammatory markers, and androgen excess suggests a multitarget benefit profile that could translate into better cycle control, ovulatory function, and hyperandrogenic symptoms over time. The strengths of this study include its retrospective controlled design; comprehensive evaluation of endocrine, metabolic, and inflammatory parameters; and the use of ANCOVA to adjust for baseline and confounding variables. In addition to our clinical observations, the broader therapeutic role of berberine has been extensively supported by recent pharmacological and molecular studies. Emerging evidence highlights its potent pleiotropic properties, particularly in modulating metabolic homeostasis, mitigating oxidative stress, and suppressing inflammatory cascades across various pathological models (24–26). These foundational mechanistic studies provide robust support for our clinical findings, further substantiating that berberine’s multitarget pharmacological profile is highly suitable for addressing the complex endocrine and metabolic dysfunctions inherent to PCOS.

This study has several limitations. First, the retrospective observational design inherently limits definitive causal inference and increases susceptibility to selection bias. Although ANCOVA was utilized to adjust for covariates and baseline comparability was demonstrated between the groups, unmeasured confounders, such as subtle differences in patient motivation or physician preference, may have influenced both the treatment allocation and the outcomes. Furthermore, critical lifestyle and socioeconomic confounders were not systematically assessed. Specifically, precise quantitative data on dietary intake, physical activity levels, socioeconomic status, and strict adherence to the prescribed treatment were unavailable. While all patients received standard diet and exercise advice as part of routine clinical care, the absence of objective monitoring means we cannot fully exclude their potential confounding effects, particularly on anthropometric and metabolic outcomes. Therefore, the treatment effects observed should be interpreted as associations rather than strict causal relationships. Future prospective trials should incorporate validated questionnaires and strict monitoring protocols to rigorously control for these variables. Additionally, due to the retrospective nature of data collection, comprehensive baseline laboratory indicators, such as complete lipid profiles and precise liver/kidney function tests, were not systematically available for all participants and thus were not included in the analysis. Although patients with known severe hepatic, renal, or lipid disorders were excluded according to our strict criteria, the lack of exact baseline numerical data limits our ability to completely rule out these unmeasured metabolic confounders. Furthermore, it should be noted that insulin resistance in our study was evaluated using HOMA-IR. While widely accepted in epidemiological and clinical settings, HOMA-IR is an indirect surrogate marker of insulin resistance. We did not perform dynamic insulin sensitivity tests, such as the hyperinsulinemic–euglycemic clamp (the gold standard) or oral glucose tolerance tests (OGTT), which limits our ability to directly and dynamically quantify insulin action in relevant tissues. Finally, the relatively short follow-up period of 3 months restricts our ability to verify the long-term sustainability of the intervention’s effects. Furthermore, our study was specifically designed to evaluate the metabolic and endocrine changes caused by berberine. Thus, long-term reproductive outcomes such as ovulation or clinical pregnancy rates were not assessed. It should be noted that our study primarily consisted of women seeking cycle regulation and metabolic improvement, rather than those undergoing active ovulation induction therapies. Therefore, evaluating fertility outcomes was beyond the scope of this specific phase of treatment. Furthermore, this study did not perform specific subgroup analyses (e.g., stratifying by obese vs. non-obese or insulin-resistant phenotypes). Given the current sample size of the intervention group, further stratification would result in statistically underpowered subgroups, increasing the risk of type II errors and potentially yielding misleading conclusions. Future large-scale, multicenter, prospective double-blind RCTs with extended follow-up periods and inclusion of reproductive endpoints are crucial to conduct these precision subgroup analyses and comprehensively validate the value of berberine in PCOS management.

In conclusion, our preliminary findings suggest that the addition of berberine to cyclic progesterone therapy may provide beneficial effects on anthropometric, hormonal, metabolic, and inflammatory profiles in women with PCOS. However, given the retrospective and non-randomized nature of this study, these observations cannot establish definitive therapeutic guidelines. Well-designed, large-scale randomized controlled trials are required to confirm these preliminary findings and to fully elucidate the long-term clinical utility of berberine as an adjunct therapy in PCOS management.

## Data Availability

The original contributions presented in the study are included in the article/supplementary material. Further inquiries can be directed to the corresponding author.
